# Bladder sensory desensitization decreases urinary urgency

**DOI:** 10.1186/1471-2490-7-9

**Published:** 2007-06-11

**Authors:** Carlos Silva, João Silva, Helder Castro, Frederico Reis, Paulo Dinis, António Avelino, Francisco Cruz

**Affiliations:** 1 Department of Urology, Hospital S. João and Faculty of Medicine of Porto, Porto, Portugal; 2 Department of Histology and Embryology, Faculty of Medicine of Porto and IBMC of the University of Porto, Porto, Portugal

## Abstract

**Background:**

Bladder desensitization has been investigated as an alternative treatment for refractory detrusor overactivity. Most open and controlled clinical trials conducted with intravesical RTX showed that desensitization delays the appearance of involuntary detrusor contractions during bladder filling and decreases the number of episodes of urgency incontinence.

Urgency is being recognised as the fundamental symptom of overactive bladder (OAB), a symptomatic complex which recent epidemiological studies have shown to affect more than 10% of the Western population. As anti-muscarinic drugs, the first line treatment for OAB, are far from being able to fully control urgency, the opportunity to test other therapeutic approaches is created. The present work was, therefore, designed as an exploratory investigation to evaluate the effect of bladder desensitization on urinary urgency.

**Methods:**

Twenty-three OAB patients with refractory urgency entered, after given informed consent, a 30 days run-in period in which medications influencing the bladder function were interrupted. At the end of this period patients filled a seven-day voiding chart where they scored, using a 0–4 scale, the bladder sensations felt before each voiding. Then, patients were instilled with 100 ml of 10% ethanol in saline (vehicle solution) and 30 days later a second seven-day voiding chart was collected. Finally, patients were instilled with 100 ml of 50 nM RTX in 10% ethanol in saline. At 1 and 3 months additional voiding charts were collected.

At the end of the vehicle and 3 months period patients were asked to give their subjective impression about the outcome of the treatment and about the willingness to repeat the previous instillation.

**Results:**

At the end of the run-in period the mean number of episodes of urgency per week was 71 ± 12 (mean ± SEM). After vehicle instillation, the mean number of episodes of urgency was 56 ± 11, but only 4 patients (17%) considered that their urinary condition had improved enough to repeat the treatment. At 1 and 3 months after RTX the number of episodes of urgency decreased to 39 ± 9 (p = 0.002) and 37 ± 6 (p = 0.02), respectively (p indicates statistical differences against vehicle). The percentage of patients with subjective improvement after RTX and willing to repeat the instillation at a later occasion was 69%.

**Conclusion:**

In OAB patients with refractory urgency bladder desensitization should be further investigated as an alternative to the standard management. Additionally, the specific effect of RTX on TRPV1 receptors suggests that urothelium and sub-urothelial C-fibers play an important role to the generation of urgency sensation.

## Background

Resiniferatoxin (RTX), the ultrapotent capsaicin analogue, was shown to desensitize TRPV1, a non-specific calcium channel, which is abundantly expressed in neuronal and non-neuronal bladder structures, including type C-sensory fibers [1], urothelial cells [2,3] and possibly interstitial cells [4]. Following desensitization, TRPV1 becomes less responsive to further activation by RTX or similar compounds and its expression is strongly reduced, both in the urothelium [3] and in sensory nerve fibers [5]. In addition, experimental studies have shown that desensitization reduces bladder response to distension, as shown by the increase of the volume threshold to reflex voiding [6] and decreased activation of sensory driven spinal cord genes as c-*fos *[7].

Urgency is being recognised as the fundamental symptom of overactive bladder (OAB), a symptomatic complex which recent epidemiological studies have shown to affect more than 10% of the Western population [8]. Unfortunately, anti-muscarinic drugs, the first line treatment for OAB, although providing some improvement, are far from being able to fully control this bothersome lower urinary tract symptom [9]. This creates the opportunity to test other therapies that, if promising, can be offered as second line options.

Bladder desensitization has been exploited in the last decade as an alternative treatment for refractory detrusor overactivity [10,11]. Most open [12-15] and controlled [16-18] clinical trials conducted so far showed that the number of episodes of urgency incontinence in patients with detrusor overactivity was decreased by intravesical RTX at the same time at which the compound delayed the appearance of involuntary detrusor contractions during bladder filling and increased bladder capacity. Furthermore, in a controlled study, the global urinary condition improved in 62% of detrusor overactivity (DO) patients treated with intravesical RTX but only in 21% of those that received the vehicle solution alone [18]. The effect of RTX on urgency was also reported in OAB patients without DO. In a small open label study RTX showed a trend to decrease the number of episodes of urinary urgency [19].

The present work was designed to further explore the effect of bladder desensitization on urinary urgency. As a primary objective it was investigated if intravesical RTX could reduce the number of episodes of urinary urgency. As a secondary objective the effect of bladder desensitization on urgency incontinence and urinary frequency was also analysed. This study was presented in part in abstract form [20].

## Methods

Twenty-three patients (7 males and 16 females with a mean age of 50 years, range 21–77) with OAB refractory to antimuscarinics were enrolled during 2005. All patients had at least 7 urgency episodes per week. Six patients had a neurogenic cause for OAB symptoms whereas the remaining patients were idiopathic. All gave written informed consent approved by the Ethics Committee of our Institution to receive intravesical RTX. The following exclusion criteria to enter the study were observed: age less than 18 years, pregnancy, known cardiovascular, renal, hepatic or psychiatric disorders, malignant diseases, duration of the OAB syndrome less than 12 months and concomitant bladder diseases, namely lower urinary tract symptoms suggestive of bladder outlet obstruction and/or treatments influencing bladder performance. In addition, patients were excluded in the presence of abnormal haematological and biochemical blood tests, abnormal kidney and bladder ultrasound and a positive urine culture.

The design of the study was the following. At the first visit the patients were enrolled in the study and entered a run-in period of 30 days during which anti-muscarinic drugs or other drugs that could affect bladder function were stopped. Patients filled a voiding chart of the last 7 days of this period. At the second visit the voiding chart was collected and patients were instilled during 30 minutes with 100 ml of the vehicle used to prepare the RTX solution, 10% ethanol in saline. The patients were then sent home for another 30 days. In the last seven days of this period patients filled another voiding chart. At the third visit the voiding chart corresponding to the vehicle period was collected and 100 ml of a 50 nM RTX solution were slowly instilled into the bladder by gravity and left in contact with the mucosa during 30 minutes. If detrusor contractions occurred, the solution could freely reflux into the container and then return into the bladder once the contractions wanned. The patients were sent home at the end of the treatment. Two additional visits were scheduled at 1 and 3 months after RTX instillation. At the end of each period, 7 day voiding charts were collected. At the end of the vehicle and 3 months period patients were asked to give their subjective impression about the outcome of the treatment and about the willingness to repeat the previous instillation. During the duration of the study no urodynamic studies were performed as the definitions of urgency and OAB are purely clinic.

A validated scale to assess urinary urgency is not yet available in the Portuguese language. In addition, the distinction between the words urge and urgency, possible in the English language, has no equivalent in Portuguese. Therefore, the suggestion for describing urgency according to the circumstances during which it was experienced and by the impact it had on concomitant activity being carried by the patients was followed to build up a scale easily understandable by the patients [21]. The options are listed below and the system score was taught to the patients at the first visit. Only options 3 and 4 were considered as equivalents of urgency. Option 2 was used to refer urge.

0 – "I voided because it was convenient although I did not feel a bladder sensation".

1 – "I voided because I had the sensation that my bladder was becoming full and the opportunity to void was convenient".

2 – "I voided because I had the sensation that my bladder was extremely full. I looked for a convenient place to void without feeling any eminent risk of urine leakage".

3 – "I voided because I had a sudden strong desire to void which I felt that could cause an urine leakage. I immediately interrupted what I was doing and looked for a convenient place where I started voiding in time".

4 – "I had a sudden strong desire to void that I felt that could cause urine leakage. Although I immediately looked for a convenient place to void, I had a leak before starting voiding".

RTX was obtained from Sigma and a 10 mM stock solution in pure ethanol was prepared and kept in the dark at 4°C in a glass container. For each instillation 100 ml of a 50 nM solution using 10% ethanol in saline as vehicle was prepared by one of the authors by adding 0.5 ml of the stock solution to 90 ml of saline and 9.5 ml of pure ethanol. Instillation was carried out within 30–60 minutes after preparation to minimise RTX absorption by plastic devices. Instillations of the vehicle solution and of the RTX solution were carried on without any form of analgesia or bladder anesthesia. Oral ciprofloxacin was administered for urinary infection prophylaxis at each instillation.

Urgency events, which corresponded to micturitions scored as 3 or 4, were counted for each patient at the end of the run-in period, vehicle period and at 1 and 3 months after RTX instillation. The number of patients in whom the number of urgency episodes decreased 25% or more after vehicle instillation and RTX was counted. Patients who considered to have had a subjective improvement at the same time points and would repeat instillations if necessary at a later occasion were also counted. The number of micturitions per week was obtained from the micturition charts. Data are presented as mean values per week ± standard error of the mean (SEM). Those at the vehicle period and at 1 and 3 months after RTX instillation were compared by a two-tailed paired t-test for means. Percentage of patients with ≥25% improvement in urgency episodes and with subjective improvement after vehicle and RTX treatment was compared by a z-test. A p < 0.05 was considered statistically significant.

## Results

**Figure 1:**
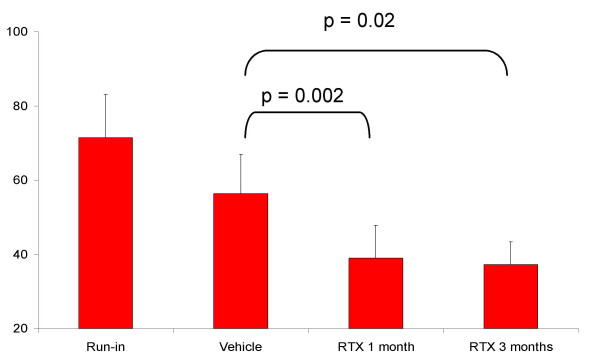
Number of episodes of urgency at the run-in period, after the instillation of the vehicle solution and at 1 and 3 months after 50 nM RTX instillation.

**Figure 2:**
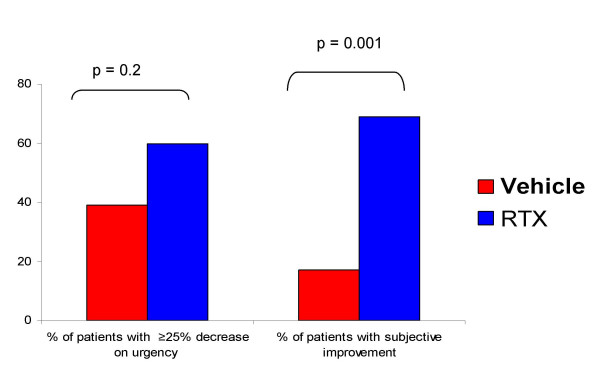
Percentage of patients with a 25% or more decrease on the number of episodes of urgency per week and percentage of patients with subjective improvement and willing to repeat the instillation if necessary, after vehicle (red bars) or 50 nM RTX instillation (blue bars).

At the end of the run-in period the mean number of episodes of urgency per week, that is micturition events preceded by sensations scored as 3 or 4, was 71 ± 12. The average number of episodes of urgency after vehicle instillation was 56 ± 11. Nine patients (39%) had a decrease in the number of episodes of urgency equal or superior to 25%. However, only 4 patients (17%) considered that their urinary condition had improved enough to repeat the treatment. The average number of urgency episodes at 1 and 3 months after RTX instillation was 39 ± 9 (p = 0.002 against vehicle) and 37 ± 6 (p = 0.02 against vehicle), respectively (Fig. 1). After RTX a 25% or more decrease over the number of episodes of urgency counted at the end of the vehicle period occurred in 14 patients representing an increase in the percentage of RTX responders to 60%. However this variation should be taken as a trend since it did not achieve statistical significance (p = 0.2). The percentage of patients with subjective improvement after RTX and willing to repeat the instillation at a later occasion increased to 69%. (p = 0.001, Fig. 2).

The episodes of urgency associated with incontinence were evaluated separately. At baseline the mean number was 21 ± 5. They decreased to 12 ± 4 after placebo instillation. At 1 and 3 months after RTX the episodes of urgency incontinence further decreased to 8 ± 3 and 9 ± 3, respectively. However, these values were not statistically different from those after vehicle instillation (p = 0.05 and p = 0.1, respectively).

**Figure 3:**
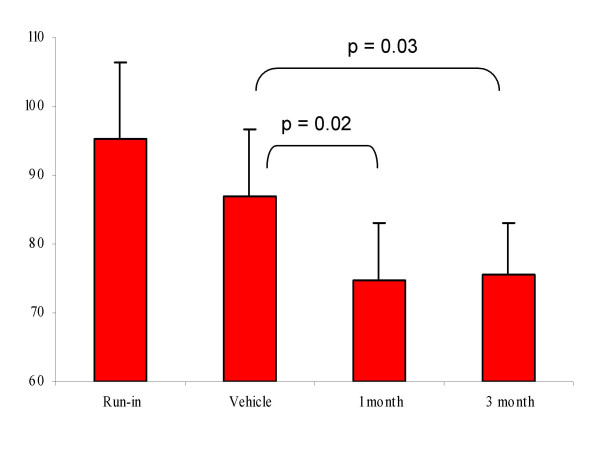
Number of micturition episodes per week at the run-in period, after the instillation of the vehicle solution and at 1 and 3 months after 50 nM RTX instillation.

The number of micturitions per week, which was 95 ± 11 at the run-in period, decreased to 87 ± 10 after the vehicle instillation. A further decrease to 75 ± 8 at 1 month (p = 0.02 against vehicle) and to 75 ± 7 at 3 months (p = 0.03 against vehicle) was observed after the RTX instillation (Fig 3).

RTX instillation was associated with a slight discomfort, described by patients as an itch sensation or urgency to urinate. In no case was analgesic medication required or instillation interrupted. After RTX treatment none of the patients reported difficulties in emptying the bladder.

## Discussion

The most important finding of the present study was the decrease of the number of episodes of urinary urgency induced by bladder desensitization. Urinary frequency also decreased and urgency incontinence showed a strong trend to a reduction. These objective changes were accompanied by a subjective improvement in more than two thirds of the patients. In addition, this study further confirmed the reports of good tolerability of RTX instillation in low concentrations [13,16]. In spite of the fact that no bladder anaesthesia was performed, all our patients carried out the 30 minutes RTX instillation to the end without difficulty.

This study was exploratory and was not designed as a randomized double arm placebo controlled trial due to the limitations in recruiting patients for an off-license treatment in one single center. Nevertheless mean episodes of urgency and micturition occurring 1 and 3 months after RTX instillation were significantly less than those after the simple instillation of the vehicle solution. In addition, RTX brought a 25% improvement in the number of episodes of urgency over the vehicle period to 60% of the patients whereas vehicle application alone had caused a similar improvement rate over the run-in period in only 39% of the patients.

Due to the lack of validated scales to quantify urgency in the Portuguese language we had to build-up a scale to assess this symptom. As the words urge and urgency have no equivalent in the Portuguese language we preferred a scale which evaluated the circumstances in which urgency occurred and its impact on the patients' daily activities, rather than its intensity. Although our option might be object of criticism, actually, the best mean to evaluate and measure urgency is not yet established and is under intense controversy. Researchers exist who prefer scales in which different degrees of urgency intensity are contemplated [22,23]. However others only recognise the necessity of distinguishing urgency form urge and consider that urgency, being a sudden and compelling desire to urinate, does not have degree of severity [24].

The reason for the improvement associated with RTX should be explained by its specific affinity to TRPV1 [25,26]. This receptor was recently found to be over expressed in the trigone of patients with urgency [27]. In C-fibers RTX binding to TRPV1 causes a massive inflow of calcium and other ions into the fiber, generating action potentials and releasing neuropeptides from peripheral nerve endings [25,26], both events contributing to itch or urgency sensations reported by some patients in the present and in previous studies in which RTX instillation was carried out [14,16]. A transient reduction of bladder sensory input conveyed to the central nervous system in C fibers then follows [7]. The interruption of C-fiber influx may thus have contributed to the symptomatic improvement observed after RTX instillation in our patients.

It is probable that part of sensory input conveyed in C fibers is not initiated by a direct stimulation of peripheral nerve endings but results from their activation by neurotransmitters and neurotrophic factors released from the urothelium [28]. RTX binding to TRPV1 present in urothelial cells may, therefore, have contributed to urgency improvement by disrupting the cross-talk between urothelial cells and suburothelial C-fibers. Inflow currents occur in human urothelial cells after TRPV1 activation suggesting that the receptor in the urothelium retains properties similar to those described in sensory neurons [29]. Likewise RTX application is followed by a reduction in urothelial TRPV1 expression [3]. Thus, RTX treatment might have reduced the potential of urothelial cells to release compounds which are known to excite sub-urothelial sensory fibres [2]. One of these compounds might be nerve growth factor (NGF) [30]. Although it is unclear at the moment if TRPV1 excitation enhances NGF release from urothelium, this neurotrophin was shown to induce bladder overactivity in experimental animals [31] and was found in high amounts in the urine of OAB patients [32].

It is also possible that RTX has prevented the ATP released from urothelial cells [2] and consequently the activation of sub-urothelial C-fibers expressing P2X3 receptors [15]. It is known that TRPV1 is involved in ATP release from the urothelium in response to stretch stimuli [33]. In addition, animal studies demonstrated that P2X3 receptors are essential for the generation of bladder contractions and noxious sensations [34]. As a matter of fact, knocking-out P2X3 in mice renders the bladder hypoactive and the animals less reactive to pain [34]. Intravesical RTX decreases both TRPV1 in urothelial cells [3] and the number of suburothelial P2X3 expressing fibers in the human bladder [15].

In our study the instillation of 10% ethanol in saline alone caused a marked improvement in the number of urgency episodes when compared to the run-in phase. It seems, however, improbable that such effect was due to a desensitizing action of ethanol on TRPV1 receptors. In fact, although ethanol was found to bind TRPV1, it does not cause its desensitization [35]. In addition, in a recent clinical trial with patients with neurogenic detrusor overactivity 10% ethanol in saline alone did not cause any variation on urodynamic parameters in contrast with the solution containing RTX 50 nM that significantly increased the bladder volume to first involuntary detrusor contraction and maximal cystometric capacity [16]. Similar findings were reported by Kuo et al. In 54 patiens with refractory DO, 21% of the patients treated with 10% ethanol improved at 3 months, a number considerably smaller that the 62% that improved after RTX 10 nM, four weekly instillations [18]. Thus, the improvement induced by 10% ethanol instillation in the present study should be taken as part of a placebo effect, a commonly recognized phenomenon in clinical trials involving OAB patients [36].

## Conclusion

This exploratory study indicates that desensitization may be useful to treat patients with urinary urgency, particularly if refractory to the standard management. This finding also has important pathophysiological implications as it indicates that C-fiber input plays an important role to the generation of the urgency sensation. Since at present RTX is the only compound with desensitizing effect suitable for human use, a randomised placebo controlled study is justified to further investigate the role of desensitization in the treatment of urgency. The dose for intravesical RTX used in the present work should be considered as indicative in future studies. In fact, all studies in which RTX was used to treat OAB symptoms [14,18,19], including the present one, used RTX concentrations of 50 nM or lower. In addition, urodynamics studies should be included in future studies. Although it was not the case of this study, urodynamics might elucidate if urgency in patients with or without detrusor overactivity have the same origin.

## List of abbreviations

ATP – adenosine triphosphate

DO – detrusor overactivity

NGF – Nerve Growth Factor

OAB – Overactive bladder

P2X3 – P2X family of ATP-gated ion channels, subtype 3

RTX – Resiniferatoxin

TRPV1 – Transient receptor potential vanilloid subfamily 1

## Competing interests

The author(s) declare that they have no competing interests.

## Authors' contributions

CS designed the study, was involved in clinical assessment of patients, analysed the data and wrote the manuscript.

CS, JS, HC, FR and PD selected, treated and followed the patients.

AA prepared the resiniferatoxin solutions.

FC participated in the design of the study and its coordination, analysed the data and wrote the manuscript.

All the authors read and approved the manuscript.

## Pre-publication history

The pre-publication history for this paper can be accessed here:


